# Parkinson’s disease: a scoping review of the quantitative and qualitative evidence of its diagnostic accuracy in primary care

**DOI:** 10.3399/BJGP.2023.0409

**Published:** 2024-03-19

**Authors:** Adnan Z Khan, Deepthi Lavu, Richard D Neal

**Affiliations:** APEx (Exeter Collaboration for Academic Primary Care), Department of Health and Community Sciences, Faculty of Health and Life Sciences, University of Exeter, Exeter.; APEx (Exeter Collaboration for Academic Primary Care), Department of Health and Community Sciences, Faculty of Health and Life Sciences, University of Exeter, Exeter.; APEx (Exeter Collaboration for Academic Primary Care), Department of Health and Community Sciences, Faculty of Health and Life Sciences, University of Exeter, Exeter.

**Keywords:** diagnostic accuracy, movement disorder in primary care, Parkinsonian symptoms, Parkinson’s disease, primary health care

## Abstract

**Background:**

Parkinson’s disease is a multisystem condition that usually presents as a movement disorder in clinical practice. There is no objective method for its diagnosis and therefore the current diagnostic process is based on characteristic clinical signs and symptoms. As the presenting symptoms can be vague and non-specific, there is often a delay in diagnosis leading to mismanagement and delayed treatment initiation. In the UK, GPs identify and initially assess individuals with Parkinson’s disease and refer them to specialists for formal diagnosis and treatment initiation.

**Aim:**

To use a scoping review to examine the available evidence on the accuracy of Parkinson’s disease diagnosis in primary care and to assess the potential for GPs to make a diagnosis and initiate treatment, and hence avoid harmful delays.

**Design and setting:**

The scoping methodology as proposed by Westphaln and colleagues that is a modified version of Arksey and O’Malley’s original framework was followed. All findings were reported according to PRISMA guidelines for scoping reviews.

**Method:**

Four databases (EMBASE, PubMed Central, Cochrane, and CINAHL) and references lists of relevant published literature were systematically searched for all types of literature available in English on the accuracy of Parkinsonism or Parkinson’s disease diagnosis in primary care. There were no search restrictions placed on countries, type of studies, or age. Two reviewers independently screened titles and abstracts followed by full-text screening.

**Results:**

Out of 1844 studies identified, only six studies met the inclusion criteria. Five were from high-income and one from a middle-income nation. Of these, three studies identified significant knowledge gaps of GPs in diagnosing Parkinson’s disease using a questionnaire-based assessment. Delay in appropriate referral because of delayed symptom identification was reported in one study. Only one study compared the accuracy of primary care Parkinson’s disease diagnosis with that of specialists, and reported that, although specialists’ diagnosis showed more sensitivity, GPs had higher specificity in diagnosing Parkinson’s disease. However, this study was found to have methodological issues leading to bias in the findings.

**Conclusion:**

This scoping review shows that there are no well-conducted studies assessing the accuracy of Parkinson’s disease diagnoses when made by GPs. This calls for more focused research in this area as diagnostic delays and errors may lead to potentially harmful but preventable delays in treatment initiation resulting in decreased quality of life for individuals with Parkinson’s disease.

## Introduction

Parkinson’s disease is a common neurological condition affecting 1–3% of the >65 years of age population.^1^ Parkinson’s disease has multifactorial aetiology with mounting evidence related to genetic risk factors.^1^^,^^2^ The hallmarks of idiopathic Parkinson’s disease are rigidity, bradykinesia, asymmetry, and a characteristic resting tremor. It is now being increasingly recognised that some non-motor symptoms such as constipation, anosmia, sleep disturbances, and depression are present for several years before the onset of motor symptoms.^3^^–^5 When assessing an individual in primary care all the above symptoms may present as vague non-specific solitary symptoms leading to a potential delay in identifying the condition early.^6^ This is in spite of the increasing awareness of the prodromal symptoms of Parkinson’s disease among clinicians. In the UK, following the initial recognition of potential Parkinson’s disease by a GP based in primary care, an individual is referred to a neurologist or specialist with experience in Parkinsonian syndromes for formal diagnosis and initiation of a management plan for the condition.^7^

Despite advances in neuroimaging and understanding Parkinson’s disease at a molecular level, there is no objective method of diagnosing this condition and its diagnosis remains largely clinical.^8^ There are no approved biomarkers for Parkinson’s disease diagnosis, and neuroimaging, at best, can only provide clues for confirming or modifying a potential diagnosis in difficult presentations. Magnetic resonance imaging (MRI) and positron emission tomography (PET) scans of the brain can detect some abnormalities and a dopamine uptake or dopamine transporter (DAT) scan may help in diagnosing patients with difficult cases.^9^^–^11 The National Institute for Health and Care Excellence (NICE) recommends use of a DAT scan only in selected cases to distinguish atypical tremors from Parkinson’s disease and both MRI and PET scans may be useful in diagnosing some ‘Parkinson plus’ syndromes and vascular Parkinsonism.^7^^,^^10^ Often only atypical tremors and tremor-only presentations of Parkinson’s disease are the ones that require additional diagnostic work-up, such as imaging or genetic testing, as suggested in several case reports.^10^

**Table:** How this fits in

There is a gap in current understanding about the accuracy of primary-care-initiated Parkinson’s disease diagnosis. This scoping review explores the literature available on the diagnosis of this condition by GPs and its accuracy. Identifying potential areas for improvement can lead to interventions for early recognition of symptoms and early diagnosis resulting in improved quality of care for patients.

The UK Parkinson’s Disease Society Brain Bank Clinical Diagnostic Criteria (UKBBC), the use of which is recommended by NICE guidance for assisting diagnosis of Parkinson’s disease, is primarily based on the characteristic motor signs of Parkinson’s disease without the use of objective biomarkers or imaging.^8^ More recently the International Parkinson and Movement Disorder Society developed the Movement Disorder Society (MDS) criteria that are comprehensive, incorporate prodromal symptoms, and highlight red-flag symptoms that, if present, point towards an alternative diagnosis.^12^ A detailed exploration of differences between the two diagnostic criteria is beyond the scope of this paper; however, a study reported that MDS criteria had 92.6% diagnostic accuracy compared with 86.4% for the UKBBC.^13^ Both criteria are based on clinical findings as there are no peripheral blood biomarker or radiological criteria for confirmation of Parkinson’s disease diagnosis.

Despite the extensive availability of clinical knowledge regarding Parkinson’s disease there is a considerable gap in current understanding of its presentation, degree of translation of clinical knowledge to aid its early diagnosis, and primary care referral thresholds. It is unknown whether early diagnosis and treatment initiation, for control of motor symptoms or for neuroprotection, would translate into slowing disease progression, improve quality of life, or overall survival. It is unclear how well GPs diagnose and refer individuals with Parkinson’s disease and how this correlates with a formal diagnosis made in secondary care. To explore this issue, the authors of the current study performed a scoping review with an aim to understand the diagnosis of Parkinson’s disease in primary care settings and to estimate the accuracy of primary care Parkinson’s disease diagnosis weighed against a gold-standard specialist diagnosis.

## Method

### Overview

A systematic search was undertaken to identify literature on the diagnosis of Parkinson’s disease in primary care. A scoping review was then undertaken to ascertain the existing knowledge base and to identify knowledge gaps for future studies and interventions. The scoping review was done in accordance with the Preferred Reporting Items for Systematic Reviews and Meta-Analyses Extension for Scoping Reviews guidelines.^14^ The methodology proposed by Westphaln and colleagues, which is a modification of original scoping review research guidelines by Arksey and O’Malley, was followed.^15^^,^^16^

### Search strategy and selection criteria

Four health databases were searched: EMBASE, PubMed Central, Cochrane, and CINAHL using combinations and variants of search terms relating to diagnosis of Parkinson’s disease, its symptoms, and primary care (Supplementary Information S1). All databases were searched on 7 November 2022 for articles published over the past 30 years (1992 to date) and the search was updated on 30 June 2023 to look for papers published since the initial search. Reference lists of published review articles were searched for additional relevant studies not identified in the database search. Experts in the field were contacted for publications not already identified.

No restrictions were placed on the publication format to widen the base of literature to be reviewed on the topic; all journal articles including reviews, guidelines, and correspondence were included in the search. Publications from all countries were included but the language was restricted to English to avoid errors arising from incorrect translation. Publications were considered eligible for inclusion if any primary care service, such as family care physicians or general practice, was involved in the interpretation of the presentation of Parkinson’s symptoms leading to a formal specialist diagnosis of the condition. There were no restrictions on age placed when including the studies. Studies where a formal diagnosis of Parkinson’s disease was missing were not included in the review. Publications also purely discussing symptoms with no diagnostic aspect or discussing only treatment options for Parkinson’s disease were not included in the review.

### Publication selection

Results of the search were imported into Rayyan — software for collaborative work on scoping studies and systemic reviews — and duplicates were deleted.^17^ Two researchers (the first and second authors) independently screened the titles and abstracts, and then carried out the full-text reviews. Reference lists of relevant publications were manually searched. Conflicts arising from uncertainty regarding the studies meeting inclusion criteria were resolved through discussion among the researchers. Where full texts of relevant publications were unavailable, attempts were made to contact the authors via email for copies of their work.

### Quality assessment

A formal quality assessment of the studies included was not performed as this was a scoping review of the available evidence. However, it is acknowledged that studies included in this review have biases because of design issues; these are discussed in detail later.

## Results

### Data extraction

**Figure 1.**
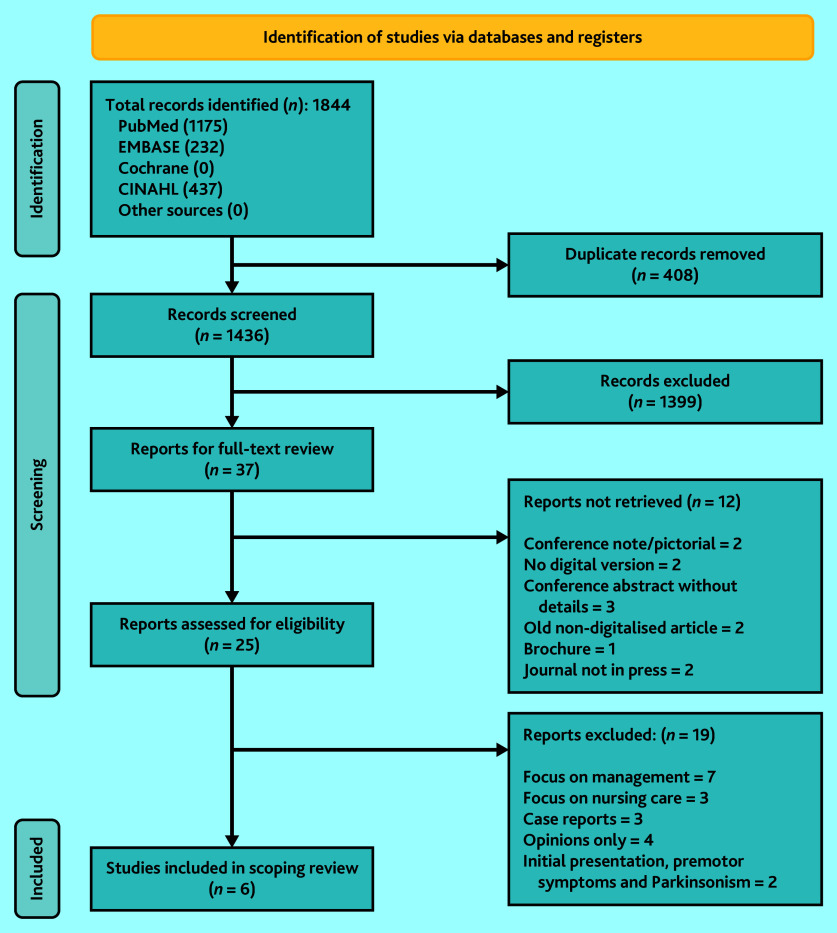
PRISMA flow chart of screening process.

The search yielded 1844 articles (1844 from the four databases, 0 from other sources) and 1436 remained after removal of duplicates. Following title and abstract screening a further 1399 articles were removed. Full texts screening of the remaining 37 articles was attempted; however, 12 articles were not retrievable, resulting in screening of the remaining 25 articles (Figure 1).

Of the 25 articles, 19 were excluded, leaving six articles that were included in the final review (Table 1).^18^^–^23 Most of the articles came from high-income countries: two from UK and one each from Singapore, Australia, and the US and only one from a middle-income country, Mexico. All were primary research articles. Three of the articles assessed the difference in accurately diagnosing Parkinson’s disease among a cohort of GPs following an intervention whereas two others analysed the accuracy of the diagnosis based on data obtained from patients diagnosed with Parkinson’s disease. The sixth study conducted in Mexico reported the results of a Parkinson’s disease patient survey. All studies were cross-sectional and lacked a control group.^18^^–^23 Key features of the studies are summarised in Table 1.

**Table 1.** Characteristics of selected studies (in chronological order by year of publication)

**Authors, study year**	**Country**	**Aim of study**	**Methodology**	**Participants**
**Meara *et al* (1999)^18^**	UK	To study the diagnostic accuracy for Parkinsonism and Parkinson’s disease in a community-based sample of participants who were on anti-Parkinsonian medication	Cross-sectional	402 patients with Parkinson’s disease
**Schrag *et al* (2002)^19^**	UK	To assess the validity of a clinical diagnosis of Parkinsonism in the general population	Cross-sectional	202 patients with Parkinson’s disease
**Tan (2007)^20^**	Singapore	To study the GP’s awareness of atypical features in early Parkinson’s disease and alternative diagnoses for Parkinsonism	Survey, cross-sectional	41 GPs
**Abbott *et al* (2011)^21^**	Australia	To assess the baseline level of knowledge and confidence among GPs in diagnosing and managing Parkinson’s disease	Interventional, pre- and post-intervention groups	105 GPs
**Thompson *et al* (2013)^22^**	US	To determine primary care practitioners’ gaps in the knowledge about Parkinson’s disease	Pre- and post-treatment impact assessment	104 GPs
**Sarabia-Tapia *et al* (2020)^23^**	Mexico	To identify the most common causes of delay in the diagnosis of the disease	Semi-structured interview, cross-sectional	112 patients with Parkinson’s disease

Findings are presented here, grouped into four key outcomes:

- diagnostic criteria;
- knowledge gap;
- time to diagnosis; and
- accuracy of diagnosis.

### Diagnostic criteria

Only one study, based in the UK, mentioned the diagnostic criteria used by the clinicians, which was the UKBBC.^18^ In all other studies the diagnosis of Parkinson’s disease was based on typical clinical features observed in the individuals and the clinical judgement of the physician involved.

### Knowledge gap

Three studies explored the area of knowledge gaps in primary care physicians about diagnosis, atypical symptoms, and management of Parkinson's Disease.^20^^–^22 All three, based on a questionnaire-based assessment, concluded that primary care physicians lack sufficient knowledge to recognise this condition early and appropriately manage it. Two studies, based in Australia and the US, also included a knowledge-based intervention to understand its impact on improving the clinician’s ability to diagnose this condition.^20^^.^^21^ The multicentred Australian study assessed the appropriate identification of motor and non-motor Parkinson’s disease symptoms among clinicians and, interestingly, found only 50% correct responses on baseline assessment.^21^ This was followed by an intervention in the form of an interactive seminar by a neurologist with special interest in Parkinson’s disease that was supplemented by written material and clinical guidelines. In the study conducted in Singapore, the participants had to attend a symposium on Parkinson’s disease and movement disorders.^20^ Both of these two studies reported statistically significant improvement in knowledge gap when checked immediately following the above interventions.^20^^,^^21^

### Time to diagnosis

The study conducted in Mexico demonstrated that poor Parkinson’s disease knowledge in primary healthcare providers resulted in a mean diagnostic delay of 2 years in 32.1% of patients.^23^ There was a mean duration of 39.3 months from the onset of symptoms to final diagnosis and this was attributed to both patient- and physician-related factors. It was noted that patients often delayed seeking medical help in the first instance with beliefs of possible spontaneous symptom remission, shame, and stigma attached to a potential diagnosis and issues related to access to specialised services being the major factors playing a role in delayed health-seeking behaviour. In the 46% of the patients who had approached their GPs with their symptoms, lack of GP’s knowledge and erroneous medical diagnosis were believed to be the main causes of further delay. The authors concluded that, in addition to early recognition of symptoms by GPs, increasing patients’ awareness and better access to information is crucial to prevent delays in diagnosis and treatment.

### Accuracy of diagnosis

Two studies, both of which were undertaken in the UK, examined the accuracy of a diagnosis of Parkinson’s disease.^18^^,^^19^ The first study, which was conducted in South Wales, reported that out of 402 individuals using anti-Parkinson medicines only 53% were correctly diagnosed with probable Parkinson’s disease.^22^ A revised diagnosis of essential tremors was made in 29% of the patients and 21% had vascular-Parkinsonism with gait apraxia preceding vascular dementia, which was misdiagnosed as Parkinson’s disease. Interestingly, 59% of patients in the study were initially diagnosed and treated in primary care for their symptoms.^17^

The second study compared the diagnosis of Parkinson’s disease made in primary care with that of a specialist based on the UKBBC.^19^ It was found that the overall sensitivity of a specialist’s diagnosis for Parkinson’s disease was higher, 93.5% (95% confidence interval [CI] = 86.3 to 97.6), compared with a primary care physician’s diagnosis, 73.5% (95% CI = 55.6% to 87.1%). However, the specificity was lower for specialists, 64.5% (95% CI = 45.4 to 80.8) compared with non-specialists 79.1% (95% CI = 64.0 to 90.0) (34 of 43 patients). This resulted in an increased negative predictive value for non-specialists compared with specialists (79.1% versus 76.9%).^19^

## Discussion

### Summary

This review is the first study, to the authors’ knowledge, that has attempted to scope the body of literature available on the diagnosis of Parkinson’s disease in a primary care setting.

Most of the studies in this review attempted to identify the knowledge profile of primary care physicians around the initial diagnosis and management of Parkinson’s disease using assessment tools with a focus on education and continued professional development. All these studies, in their pre-intervention assessments, found that GPs lack sufficient knowledge on the early signs and symptoms of Parkinson’s disease, and its initial assessment and management.^20^^–^22 Interestingly, all reported a positive impact of knowledge-based interventions, the long-term impact of which is largely unknown. In spite of their diagnostic importance, only one study explored the prodromal non-motor symptoms of Parkinson’s disease.^20^ Only one study discussed the sensitivity of diagnosis by clinicians and reported it as higher for specialists but acknowledged that GPs had better diagnostic specificity.^19^ Also only one study discussed the time to diagnosis and initiation of treatment, and it identified patients’ indecisiveness as an important factor in determining the diagnostic timeline.^23^ No studies investigated the impact of time to diagnosis on the treatment options and outcomes, overall wellbeing of the participants, or the degree of progression of symptoms.

### Strengths and limitations

This scoping review is the first of its kind, to the authors’ knowledge, as it explores the evidence available on diagnosis of Parkinson’s disease in primary care. Its strengths include a systematic and transparent approach to the identification of studies alongside a focus on a well-defined topic. However, there are some limitations to this review. First, as the boundary between primary and secondary care in different healthcare systems is not well defined, the authors might have inadvertently excluded some of the available studies from this review. Similarly, differences in the referral process for this condition might have precluded some studies from being included in the final review. Finally, as some studies categorised GPs and non-neurologists as specialists, it was difficult to extract information related to primary care alone.

### Comparison with existing literature

As a result of a lack of objective testing, the acceptable gold standard for diagnosis of Parkinson’s disease is a diagnosis generated by a specialist that is in line with guideline-based criteria. In the UK, as recommended by NICE, GPs refer individuals with classic Parkinson’s disease symptoms to specialist care for establishing diagnosis and treatment initiation.^7^

Outside the primary care setting, several studies have investigated the diagnostic accuracy of physicians based on input from specialised centres for movement disorders.^24^^,^^25^ A meta-analysis of 20 different studies, from across the world, performed by researchers in the US, reported an accuracy of 73.8% for clinical diagnosis suggested by non-specialists, including a wide range of doctors such as general neurologists and geriatricians, and 79.6% for that by movement disorder experts.^26^ A letter published in the *British Medical Journal* in 2006 suggested that, because of the high error rate of Parkinson’s disease diagnosis in primary care, all individuals suspected of having Parkinson’s disease should be referred to specialist care.^27^ However, no detailed information on where the error rate was obtained from was provided. The current review shows that, except for a study from the UK, no other studies to date have aimed to establish the accuracy of Parkinson’s disease diagnosis initiated in primary care.^19^ The UK study compared the diagnostic accuracy of a non-specialist, such as GPs, with that of a specialist (neurologist or a geriatrician) and, as the study was conducted before the NICE 2006 guideline, it is likely that referral for specialist review might have been made only in situations where there was diagnostic difficulty. The study concluded that GPs performed reasonably well in terms of the sensitivity of their diagnosis and had better diagnostic specificity than specialists. Importantly, the investigators acknowledged the fact that specialists had the benefit of making a diagnosis after some time had elapsed leading to more apparent clinical symptoms.

The current review also identified some studies that were community based and did not compare the diagnostic accuracy of GPs directly with that of a specialist. One of them was the study conducted in Wales, included in this review, which attempted to investigate the diagnostic accuracy of an existing Parkinson’s disease diagnosis in a community-based sample. The participants were re-examined by a movement disorder specialist and a neurologist. However the study did not mention the initial incorrect diagnosis made by a specialist.^18^ Another study, based in Scotland, examined the diagnostic accuracy of Parkinson’s disease in a community setting among a cohort of 610 individuals taking anti-Parkinson medicine.^28^ Out of that cohort, 64 consenting individuals were re-assessed by two neurologists who used PET scans for assisting with decisions on patients with difficult cases and 60.9% of them were found unlikely to have Parkinson’s disease, leading to a conclusion that there is a high error rate in primary care Parkinson’s disease diagnosis. This conclusion is, however, flawed as only 19% of the 610 individuals identified were initially diagnosed and managed in GP surgeries and the paper neither expanded on who made the initial diagnosis, nor did it explicitly compare the diagnosis made by GPs with that of the neurologists. This study was not included in scoping review as its focus was on antiparkinsonian therapy reassessment and stopping if a revised diagnosis was made.

Despite advancements in understanding the pathobiology of Parkinson’s disease, the ultimate diagnostic criteria remain clinical and there is no objective test to work as an arbiter for diagnostic uncertainty. Studies, such as DATATOP (Deprenyl and tocopherol antioxidative therapy of Parkinsonism), have shown that diagnostic uncertainty is observed even among specialists with 8.1% of participants being given an alternative diagnosis to Parkinson’s disease during a 7-to 8-year follow-up period in spite of using expert-based diagnosis for initial participant recruitment.^24^ Similarly, the HUNT study conducted in Sweden showed that the diagnostic accuracy of experts for Parkinson’s disease was only 65% and the authors concluded that the overall quality of diagnosis for Parkinson’s disease was suboptimal.^29^ These show that even specialists, not just primary care physicians, have difficulty establishing an accurate diagnosis in a large number of patients. This finding is important to acknowledge, as keeping in view the positive impact of knowledge-based intervention as reported in two studies included in this review indicates the possibility that, with minimal and targeted interventions, the sensitivity of diagnosis initiated by GPs could be enhanced.^21^^,^^22^ Such interventions have potential to reduce the time to diagnosis and enable early initiation of treatment when needed.

This review also highlighted the gap in the exploration of prodromal and non-motor symptoms for the diagnosis of Parkinson’s disease in the primary care setting. Most of the studies reviewed did not explore these symptoms; except one where anosmia was a part of the pre-intervention assessment questionnaire.^20^ It has been increasingly recognised that non-motor symptoms of Parkinson’s disease, such as anosmia, gastrointestinal symptoms, mood and sleep disturbances, start much before the onset of motor symptoms.^5^^,^^6^ A meta-analysis comparing data from seven studies found 3.84-fold risk of developing Parkinson’s disease in patients with an isolated finding of hyposmia (pooled relative risk: 3.84, 95% CI = 2.12 to 6.95).^30^ Another study found that the risk of developing characteristic Parkinson’s disease symptoms was 10% after an average of 2 years from an initial presentation of hyposmia.^31^ Most of this information is from retrospective studies based in specialised centres with minimal primary care input and highlights a gap in knowledge of prodromal symptoms that could be identified in the primary care setting to allow earlier identification of Parkinson’s disease.

### Implications for research and practice

As established from this review, there is a clear gap in knowledge regarding various aspects of diagnosis of Parkinson’s disease in the primary care setting. There are very few to no studies in primary care that have explored the prodromal symptoms of Parkinson’s disease and time to diagnosis, the accuracy of potential diagnosis by primary care professionals, and the outcomes of time to diagnosis based on initial presentation to primary care with early motor and non-motor symptoms.

The authors of the current review recommend further research into the dynamics of diagnostic processes for Parkinson’s disease in primary care. First, the diagnostic accuracy of GPs needs to be measured. This research should use a consultant’s diagnosis as the gold standard and results discussed in terms of positive and negative predictive value and a receiver operator curve. This should ideally involve the measurement of time lag from initial primary care presentation to treatment initiation. Second, GPs’ knowledge gaps in the identification of prodromal and non-motor symptoms need to be explored further. Primary healthcare utilisation for non-motor symptoms is another area that would benefit from further investigation. Findings from these could have major implications on various primary care interventions that may allow timely care for individuals with Parkinson’s disease and potentially significantly improve their quality of life.

In conclusion, there is a dearth of well-designed studies that assess the diagnostic accuracy of GPs when managing individuals with possible Parkinson’s disease. This scientific gap has become more obvious after the introduction of NICE guidance, especially in the UK healthcare system. The current review shows that no study has compared an initial GP’s diagnosis of this condition before referring to a specialist with the final diagnosis made by a specialist, both using clinical skills of history taking and examination alone as diagnostic tools. One study that is also included in this review attempted to do so retrospectively on a cohort of patients that were already diagnosed either by a specialist or a GP before the introduction of current NICE guidance.^19^ This finding highlights the neglected area of knowledge-based interventions to increase the clinical knowledge and skills of GPs about movement disorders in general and Parkinson's disease in particular. This also necessitates further studies that compare individuals’ initial symptoms and clinical signs indicating a potential diagnosis of Parkinson’s disease with the clinical parameters observed during a specialised assessment. Such interventions can potentially lead to earlier identification of Parkinson’s disease by GPs resulting in faster initiation of treatments and offering other appropriate support to patients, overall hopefully culminating in improved quality of care for individuals.

## Data

All studies are in the public domain.
